# Human Olfaction, Crossmodal Perception, and Consciousness

**DOI:** 10.1093/chemse/bjx061

**Published:** 2017-09-19

**Authors:** Barry C Smith

**Affiliations:** 1 Institute of Philosophy, School of Advanced Study, University of London, Senate House, Malet Street, London WC1E 7HU, UK

When philosophers attempt to provide a theory of perception they usually focus exclusively on vision, assuming, without argument, that it can serve as a model for perception in general, extendible, with minor modifications, to all other senses. This is a hopeless strategy and Andreas Keller’s monograph is a useful corrective to it. The concluding chapter, *Comparing Olfaction and Vision*, would be an excellent self-standing essay. In fact, it is Keller’s desire to distance himself from visuocentrism in the philosophy, psychology, and neuroscience of perception that led him to pursue a second doctorate on philosophy and write this monograph on olfactory perception.

As Keller reminds us, “visual perception in humans is fundamentally different from human olfactory perception” (p.193). Olfaction does not maintain a permanent olfactory scene in the way vision sustains our view of our immediate surroundings. There is no agreed way to organize perceptual smell space or identify its fundamental categories as there is for color or sound space. Olfaction has close ties to the emotions and only weak links to language; odors are difficult to name. The grouping of odors in olfactory perception reflects the behavioral needs and responses of individuals, not physical similarities among the stimuli. These distinguishing features give olfaction a different function and character from vision; in particular, olfactory perception does not involve, for Keller, the perception of objects.

A key reason for this conclusion is Keller’s conviction that the perceptual space of olfaction is not spatially or temporally structured. The arguments for this claim come in Chapter 3 where he discounts different attempts to establish candidates for olfactory objects. His strategy is to consider criteria for objecthood in a perceived scene and to see whether these criteria are met in the case of smells. These include a distinction between figure and ground in perception, and the so-called Many Properties problem of deciding, for any perceived scene, which properties go with which objects; an instance of the binding problem. Keller works through cases and finds them wanting. Smells may be distinct to the perceiver, the smell of baked goods and fresh coffee, say, but which is the figure and which is the ground? We seem unable to provide an answer. The trouble with this strategy is that is takes the figure-ground distinction in olfaction to be modeled on the visual case, and Keller even tells us at one point:

To decide if any of these situations [the examples given] should be considered as evidence for figure-ground segregation, it is informative to compare them to analogous situations in visual perception. (p.74)

At this point, readers may be wondering what happened to the insistence that we do not model olfactory perception on vision. The worry is that Keller has not exercised sufficient imagination in the range of cases he considers when attempting to distance himself from the visuocentric outlook. The point is that we can find temporal and spatial aspects of olfactory experiences if we look hard enough.

For example, if I am in a restaurant and someone lights up a cigarette, I have a pretty accurate idea of how near or far away they are from me. Although the smell of cigarette smoke cannot convey the direction from which the smoke emanates, it conveys an idea of the distance of the source. Similarly, I may enter a room and learn just from the character of the perceived odor that someone was smoking recently, or had been smoking a little while ago. And although most of us will have habituated to the smell of our own homes, this is not the same as not smelling it anymore. People with acquired anosmia speak of feeling alienated and cut off from their own home. By contrast, normosmics will have the (perhaps unconsciously) perceived smell of their home as a baseline against which to detect when someone has been there, or has been smoking, or left the garbage out. These perceptible changes to the baseline odor are foregrounded for the perceiver in a figure-ground way.

For the Many Properties Problem Keller provides a nice example—the smell of pizza—but he draws entirely the wrong morals and it is illustrative to see why.

When an anchovy and garlic pizza is delivered, we know that the anchovy smell and the garlic smell are both properties of the pizza smell. However, this knowledge is based on background knowledge and assumptions rather than on perceptual grouping of the anchovy and garlic smell (pp.76–77).

But why does this perceptual experience require “background knowledge and assumptions” instead of just perceptual grouping? Keller tells us that:

If the pizza that is delivered does not have anchovies on it, but the pizza delivery man carries some anchovies in his pocket to snack on, we will incorrectly assume that the garlic smell and the anchovy smell are properties of the pizza smell (p.77).

The natural response is that our perceptual experience of pizza as the object with that smell, and the source of that smell, is based not on “background knowledge and assumptions” but repeated multisensory experience of seeing, touching, smelling, and tasting pizza in which all those smells combine with other properties (in particular taste, giving rise to flavor perceptions) to provide us with the perceptual experience of eating pizza. The fact that our perceptual systems can be bluffed in odd situations into expecting the flavor of anchovies by aromas that are not part of the pizza is a small price to pay for the fact that our perceptual systems usually deliver accurate expectations and knowledge of objects.

Keller’s worry is that vision has no such limitations in grouping the right properties with the right objects—though we cannot just see whether a tomato and anchovy sauce has anchovies in it. We need our nose for that; and besides, why should vision be the right analogy here?

Keller thinks that: “our assigning of properties to smells is not based on the structure of our percepts but on assumptions about the most likely scenarios” (p.77). But this invokes an unnecessary high-level cognitive process to solve a problem that is often the result of crossmodal associations between inputs from different sensory systems and experience of how they typically combine in everyday episodes of multisensory perception. We do not just smell odors, we learn them in a context where we experience the properties of their sources. An orange odor is usually learned in the context of holding a fruit with a distinctive look and feel. Perception does not take place modality by modality in separate packets of seeing and hearing, feeling and smelling. The information that shapes perceptual experience is multimodal: and multisensory integration is the exception not the rule.

The handling of crossmodal correspondences and the multisensory nature of experience is the weakest part of Keller’s book. There is cursory mention of it and he could have done much more to recruit its resources to explain various puzzles he ponders. It is probably the violation of multisensory expectations that explains why the naming of odors is so hard. People will say an odor is familiar but struggle to find its name, and since we name odors by their sources it is the difficulty of connecting an odor to its source that makes naming so hard. The problem is confounded when sniffing a colorless liquid from a vial while trying to identify it as pineapple. Not only are the multisensory cues of the characteristic look and feel of a pineapple missing, there are counter cues about the stimulus being a colorless liquid. Not surprising, therefore that we struggle to find the name, despite having lots of information about the source; namely, that its edible and a fruit.

The problem of odors being detached from their sources illustrates a key respect in which olfaction differs from vision. A visual image or perception has as part of its semantic content a representation that resembles the object it represents, be it a face or a house; whereas an olfactory perception or image does not have in its content anything that shows what it is a perception or image of. This leads Keller, too quickly, to suppose olfactory perception cannot identify its source as object. However, when we acknowledge the help odor perception gets from the other senses, we can treat perceptual experience, including perception of smell as perception of the source of the smell.

Keller is worried that if we experience the smell of a lion we might undergo that experience not in the presence of a lion, but rather from the zoo keeper’s clothes. It would still be the smell of a lion even though we may be mistaken that there is a lion nearby if we only smelled it. Equally, we can be fooled by a chemical concoction that synthesizes the smell of a lion without any contact with lions. Again, these examples simply show that we can be mistaken but that is part and parcel of perception. It would not be a case of perceiving if it was not possible to misperceive. Our perceptual systems are not infallible.

Finally, how does smell fit into consciousness? Keller devotes two chapters of the book to addressing this question and comes up with the frequently advanced view that most of olfaction is unconscious. That is, we are not aware of smelling lots of complex odor mixtures in our environment. But the conclusion depends on a controversial philosophical thesis that consciousness requires attention; in other words, unattended, though registered, stimuli are not consciously but only unconsciously perceived. It is certainly true that people do not attend to smells unless they are intense, or have been brought to their notice as worth sniffing. However, it does not follow that smell features very little in consciousness and that they are mostly perceived unconsciously.

Keller is right when he says: “Odor stimuli induce desires, emotions, and physiological responses that make us respond to smells in automatic ways” (p.117). But they can also contribute to the quality of the experiences we have of desiring, feeing, or responding, providing there is a certain quality to those experiences that would be different in the absence or loss of our sense of smell. Instead, of thinking of smell as mostly unconscious, we could think of it as providing the background to consciousness, being unattended but modulating our experience moment to moment. The idea of conscious experiences that occur without our awareness or attention to them can be illustrated by the case of a constant toothache. All day it can plague you but there were parts of the day when you forgot about it or did not notice it, then you remembered and it was there again. Did the pain simply go away and come back when we noticed it, like the light in the fridge? Or was it going on but occasionally unattended? The latter is more likely and certainly more plausible than countenancing partly unconscious pains.

The key example for consciously experienced but unattended olfactory experiences is when they contribute to our experiences of a food’s flavor. People can eat without paying much attention, especially when eating in front of the TV, and yet, they experience the food’s flavor and would notice the absence of smell’s contribution to the flavor of what they eat. These are interesting cases where smell makes a palpable contribution to the experience of flavor and yet is not recognized as such, as smell, because it is due to retronasal olfaction and not to orthonasal olfaction—inhaling or sniffing. In his recent book, *Neuroenology*, Gordon Sheppard even goes so far as to claim that retronsal olfaction is unconscious; although the contribution to our conscious enjoyment of food certainly does not go missing in normal cases. Unrecognized and at times unattended, but the retronasal contribution of smell to tasting food, has a big conscious impact. Keller misses this because of the brief coverage he gives to olfaction’s role in multisensory flavor perception.

That said, there is much to admire in Keller’s book. It is a clear and engaging presentation of much recent research on olfaction and could help to bring the interest and puzzles of olfaction to a wider audience. The philosophy is on the whole accessible, especially in the later chapters after the idea of individuals’ perceptual quality spaces has been spelled out. These amount to the subjectivities of individual perceivers’ judgments of similarity between stimuli of any kind, within and across the modalities. The problem with such a maneuver is that everything is similar to everything else in some respects. What we need to know are the relevant respects in which smells, or even smells and tastes, are similar to one another. The rest of the book tries to spell these out and it does a very good job even if there is, as one would suspect, still more to do.

## Funding

This research is supported by AHRC Science in Culture Grant Reference - AH/N50452X/1.

